# Reliability of ultrasound in evaluating the plantar skin and fat pad of the foot in the setting of diabetes

**DOI:** 10.1371/journal.pone.0257790

**Published:** 2021-09-23

**Authors:** Troy Morrison, Sara Jones, Ryan Scott Causby, Kerry Thoirs

**Affiliations:** 1 Allied Health and Human Performance, University of South Australia, Adelaide, South Australia, Australia; 2 Department of Rural Health, University of South Australia, Whyalla Norrie, South Australia, Australia; di Pompeo d’Illasi, Universita degli Studi di Roma La Sapienza Facolta di Medicina e Psicologia, ITALY

## Abstract

Ultrasound can be used to assess injury and structural changes to the soft-tissue structure of the foot. It may be useful to assess the feet of people with diabetes who are at increased risk of plantar soft-tissue pathological changes. The aim of this study was to determine if ultrasound measurements of plantar soft-tissue thickness and assessments of tissue acoustic characteristics are reliable in people with and without diabetes mellitus. A repeated measures design was used to determine intra-observer reliability for ultrasound measurements of plantar skin and fat pad thickness and intra- and inter-observer reliability of plantar skin and fat pad tissue characterisation assessments made at foot sites which are at risk of tissue injury in people with diabetes. Thickness measurements and tissue characterisation assessments were obtained at the heel and forefoot in both the unloaded and compressed states and included discrete layers of the plantar tissues: skin, microchamber, horizontal fibrous band, macrochamber and total soft-tissue depth. At each site, relative intra-observer reliability was achieved for the measurement of at least one plantar tissue layer. The total soft-tissue thickness measured in the unloaded state (ICC 0.925–0.976) demonstrated intra-observer reliability and is the most sensitive for detecting small change on repeated measures. Intra-observer agreement was demonstrated for tissue characteristic assessments of the skin at the heel (k = 0.70), fat pad at the lateral sesamoid region (k = 0.70) and both skin and fat pad at the second (k = 0.80, k = 0.70 respectively) and third metatarsal heads (k = 0.90, k = 0.79 respectively). However, acceptable inter-observer agreement was not demonstrated for any tissue characteristic assessment, therefore the use of multiple observers should be avoided when making these assessments.

## Introduction

The plantar skin and fat pad protect the foot and provide shock absorption against the mechanical stresses experienced during gait [1]. Ultrasound imaging can characterise the anatomy and the extent of injury, structural change or pathology of the plantar skin and fat pad.

In people with diabetes mellitus (DM), the plantar tissues of the foot can undergo structural changes as a direct consequence of hyperglycaemia and non-enzymatic glycation [2] where the accumulation of advanced glycation end-products (AGEs) contribute to the pathogenesis of several diabetes-related disorders [2]. In the foot, AGE accumulation manifests as degenerative change in the connective tissue structure (collagen and elastin) and endothelial dysfunction, which is expressed as micro and macrovascular disease (secondary to wall stiffening and endothelial damage) and contributes to the development of peripheral neuropathy [2–6]. Resultant structural tissue changes include joint and soft-tissue stiffening, fat pad and skin atrophy and mechanical displacement of the sub-metatarsal head fat pads [3, 7–10], culminating in reduced mechanical efficacy [11] and structural integrity [12]. Subsequent alterations to plantar skin or fat pad thickness or elasticity reduce the foot’s ability to resist and absorb mechanical loads, setting a precedent for increased peak plantar pressure, tissue stress and accelerated tissue breakdown. The foot becomes vulnerable to ulceration, particularly in the presence of reduced joint mobility, foot deformity [6] and peripheral neuropathy [13–16]. In people with DM, the prevalence of foot ulcers is 4–10%, with a lifetime risk as high as 25% [17]. Amputation is a serious sequelae, with up to 85% of amputations preceded by ulceration [14], and a mortality rate of up to 80% at five years [17]. Regular clinical screening has demonstrated a reduction in diabetic foot complications [17, 18], and ultrasound screening has potential for a further positive impact.

The pathway to diabetes-related foot ulceration is due to a combination of two or more contributors [19]. To identify these contributors and the risk of a person with diabetes developing a foot ulcer, a comprehensive history and clinical assessment for dermatological, musculoskeletal, neurological and vascular perfusion changes is required [20]. Plantar pressure measurements (PPM) have also been used to identify foot sites of higher ulceration risk [13, 16, 21, 22]. Nonetheless, PPM and clinical risk assessments may not necessarily capture subclinical manifestations that can precede ulcer development, such as tissue degeneration, stiffening and atrophy. Ultrasound demonstrates this soft-tissue detail, and studies have suggested that ultrasound measurements of soft-tissue thickness [12, 23, 24] may have a role in assessing the risk of plantar foot ulceration in people with DM. However, the reliability of ultrasound measurements of the plantar skin and fat pad thickness are not well documented [25]. Ultrasound can also be used to depict tissue characteristics other than thickness, but such assessments are often overlooked, including those of the plantar foot in diabetic people, due to their perceived subjectivity [26–28]. Consequently, the reliability of ultrasound for describing plantar tissue characteristics in the setting of diabetes remains unknown. It is important that the reliability of such ultrasound measurements is understood before pursuing their clinical use. The aim of this study was to determine if ultrasound measurements of thickness and tissue characteristics of the plantar soft-tissue, made at foot sites at risk of tissue injury in people with diabetes, are reliable.

## Materials and methods

A repeated measures study design was used to determine the reliability for ultrasound measurements and assessments of the plantar skin and fat pad. Specifically, the intra-observer reliability for thickness measurements and the intra- and inter-observer reliability for tissue characterisation assessments. Participants for reliability testing were drawn from a larger study of 115 participants with and without diabetes conducted by the authors using the same ultrasound imaging protocol described in this study. A random number generator was used to select participants for repeated tissue characterisation assessments. This study was approved by the University of South Australia’s Human Research Ethics Committee (protocol number 000003603) and all participants gave written informed consent.

### Sample

The sample size for intra-observer measurements of thickness was determined using PASS 16 (Power Analysis and Sample Size software, NCSS, LLC. Kaysville, Utah, USA) [29]. It was estimated that to detect an intraclass coefficient (ICC) of 0.70, with two observations per participant, a minimum of 10 participants were required to achieve at least 82% power (alpha level of 0.05). A convenience sample of 15 participants with and without diabetes were recruited. Exclusion criteria were an absent dorsalis pedis pulse, significant lower extremity musculoskeletal disorder or injury, surgery to the foot or known peripheral neuropathy that cannot be attributed to diabetes.

The sample size for intra-observer and inter-observer assessments of tissue characteristics was estimated for a two-observer study on a dichotomous variable with an alpha level of 0.05, power of 80% and a two-tailed test [30]. An *a priori* sample size of 22 participants was estimated, based on the minimal acceptable kappa value of 0.60, assuming independence and no bias between observations.

### Observers

An accredited sonographer (TM) with 18 years clinical ultrasound experience and specialist experience in advanced musculoskeletal ultrasound (MSKU) performed all thickness measurements. All ultrasound tissue characteristic assessments were performed independently by two accredited sonographers: Sonographer 1 (TM) and Sonographer 2 (KT) who had 25 years clinical experience in MSKU and research.

### Equipment

Ultrasound images were produced using a Philips IU22 X-Matrix system (Philips healthcare, Bothell, WA, USA) with a 5-12MHz broadband linear array transducer (50mm aperture, 256 elements), which is designed for musculoskeletal applications and has a lateral resolution of 0.9mm at 20mm depth and 1.0mm at 40mm depth. The axial resolution at 20mm and 40mm depth is 0.7mm and 0.8mm, respectively. A factory musculoskeletal imaging pre-set (*MSK General*) was used and imaging settings such as dynamic range, persistence and frequency were consistent across participant trials. There were no other image optimisation restrictions.

### Study scan protocol

#### Thickness measurement (intra-observer)

Measurements were made from images acquired from one foot of each participant, while lying in a semi-recumbent position on an examination couch with the leg extended. The choice of left or right foot was determined in advance by randomisation. Each participant underwent two imaging trials performed by the same sonographer, each consisting of 40 measurements across four anatomical sites of the plantar foot. Real-time imaging was used to identify the measurement site and image from which measurements would be made. Measurements were made from a static image using the machine’s inbuilt electronic callipers.

All thickness measurements were made from an image acquired in the longitudinal imaging plane (relative to the foot) across four plantar foot sites; heel, lateral sesamoid of the hallux and 2^nd^ and 3^rd^ sub-metatarsal heads. These sites were selected as they represent areas known for increased peak plantar pressure and frequency of ulceration [31, 32].

**Fig 1:**
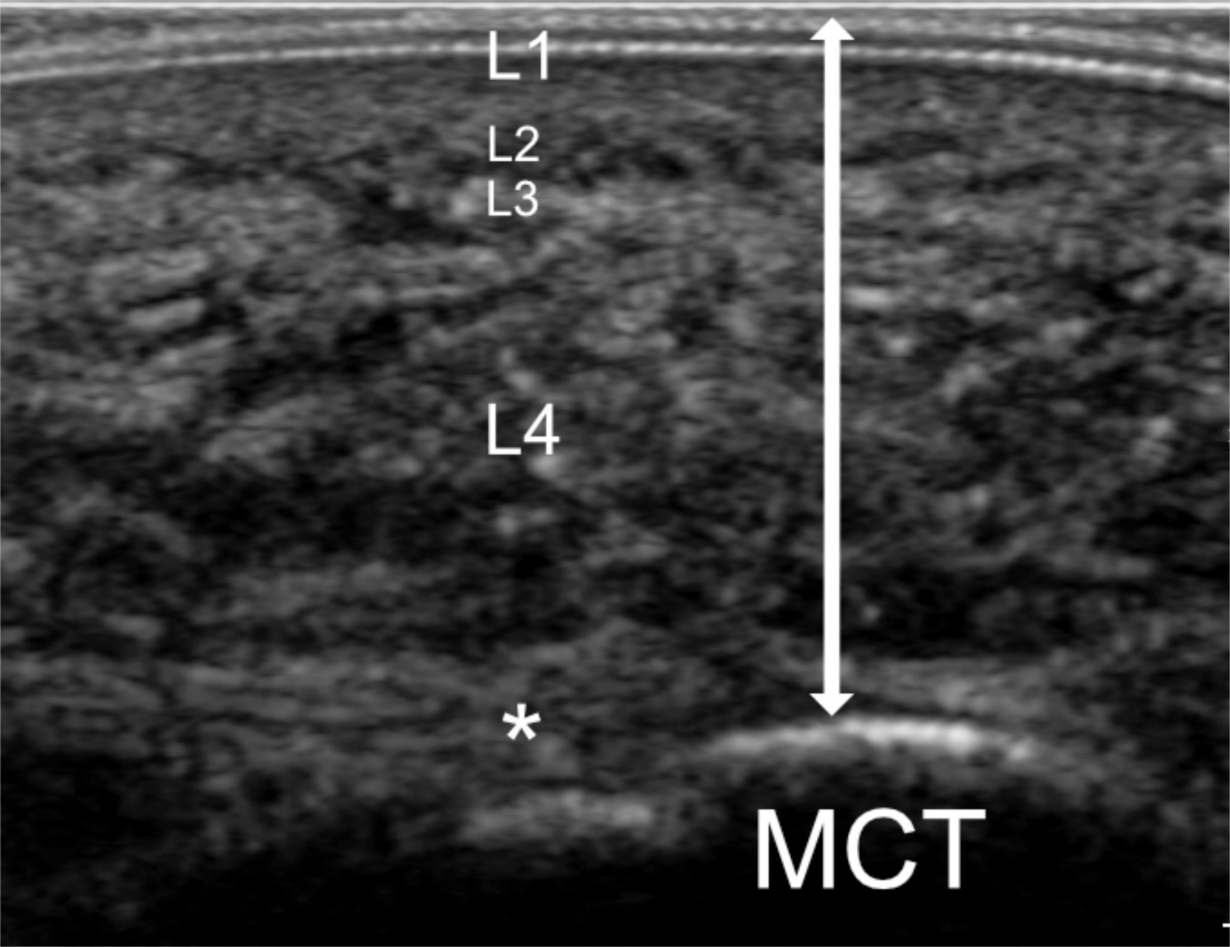
Longitudinal ultrasound image at the plantar heel demonstrating transducer positioning and measurement location (line with arrows). L1; plantar skin, L2, microchamber layer, L3; horizontal fibrous band, L4; macrochamber layer, MCT; medial calcaneal tubercle, asterisk; plantar fascia.

To acquire the image at the heel, the transducer was positioned over the plantar fascia origin at the medial calcaneal tubercle and orientated so that its long axis was aligned with the second ray, perpendicular to the skin surface. Measurements were made at a point on the image where the calcaneus was most prominent (Fig 1).

To acquire the image at the lateral sesamoid of the hallux, a transverse imaging plane (relative to the foot) was used to identify the sesamoid bones. These were identified by their typical appearances of convex and attenuating bony islands superficial to the first metatarsal head. The lateral sesamoid was recognised as the most lateral of the two bones and was positioned within the central field. The transducer was then pivoted clockwise 90 degrees, to correspond to the lateral sesamoid’s longest axis, while maintaining a perpendicular orientation to the bone. Measurements were made at a point where the curvilinear cortex was most superficial.

**Fig 2:**
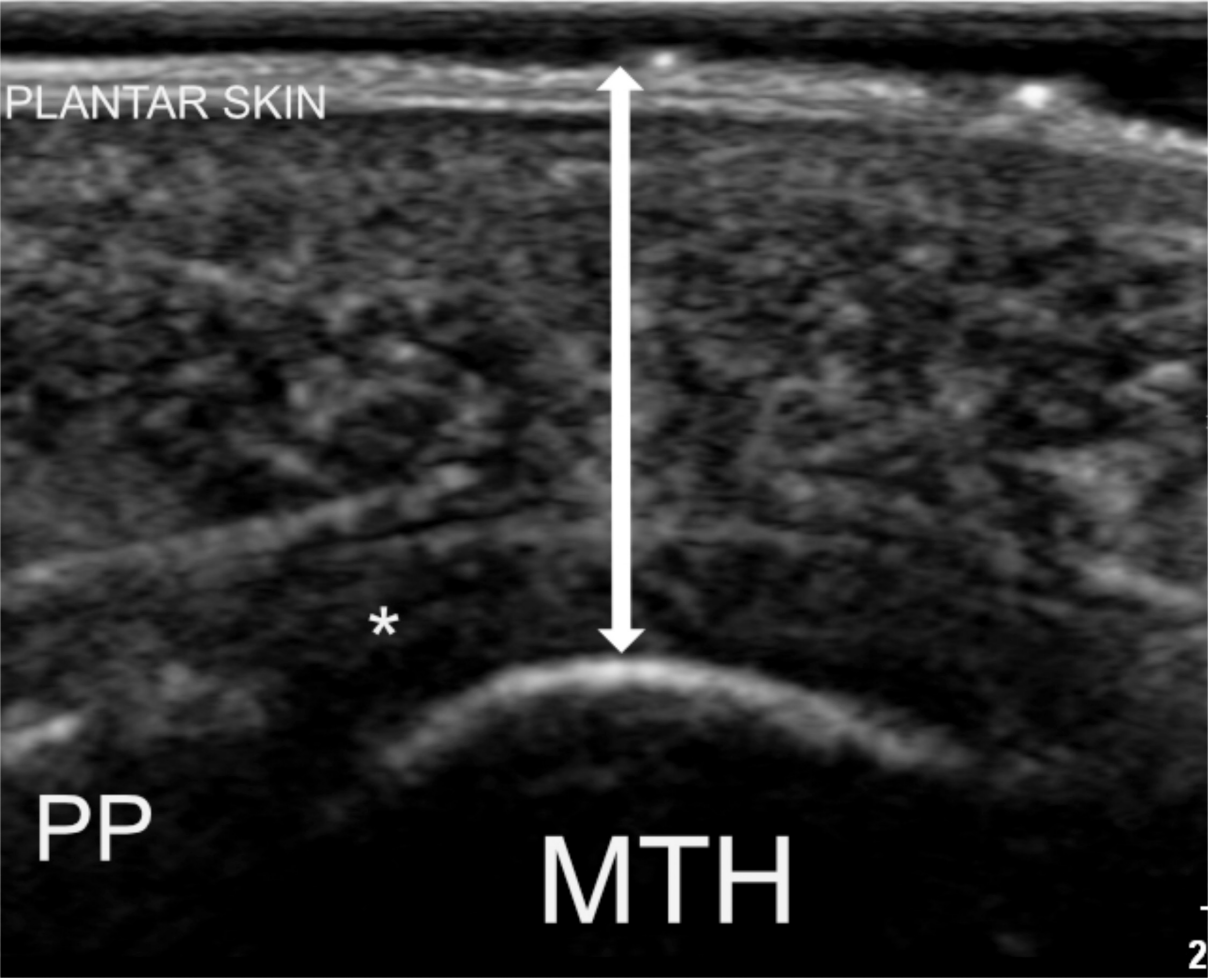
Longitudinal ultrasound image overlying the second metatarsal head demonstrating transducer positioning and measurement location (line with arrows). MTH; metatarsal head, PP; proximal phalanx, asterisk; plantar plate.

To acquire the images at the second and third metatarsal heads, the transducer was positioned over the plantar forefoot in line with the longitudinal axis of each respective metatarsal. Transducer position and tilt were adjusted to profile the mid-point of the metatarsal head in its maximal convexity with the transducer orientated perpendicular to this. Confirmation of the correct metatarsal was achieved using real-time dynamic evaluation of the corresponding toe, performed using the sonographer’s free hand. The metatarsal head was positioned in the centre of the field-of-view. Tissue thickness measurements were taken at the site of maximal cortical convexity (Fig 2).

Each measurement was made first in an unloaded state and second in a compressed state. An *unloaded state* was defined as having a layer of gel stand-off separating the skin and transducer, to avoid tissue compression or deformation due to transducer pressure. A *compressed state* was where manual vertical compression of the transducer was applied by the sonographer to cause tissue deformation. Compression was applied in real-time until the tissues within the central field-of-view, at the site of interest, were no longer deforming.

Five measurements of discrete anatomical layers were made in both the unloaded and the compressed states (10 measurements) at each of the four plantar foot sites; L1 (the skin; combined epidermis and dermis), L2 (superficial subcutaneous microchamber layer), L3 (horizontal fibrous band), L4 (deep subcutaneous macrochamber layer) and TSD (total soft-tissue depth, layers 1–4 combined).

For each participant, TM performed the imaging protocol (trial 1) and then immediately repeated the imaging protocol (trial 2) to avoid the potential confounding effects of the participant bearing weight between trials and to control the test-retest environment. Although the second data set was obtained immediately following the first, memory recall of measurements was limited due to the large number of measurements taken and the time taken to complete a full set of measurements on a single participant (approximately 20–30 minutes).

#### Tissue characteristic assessment (intra-observer, inter-observer)

All images were acquired in the unloaded state by one sonographer (TM), at the same sites and using the same methods described in the *thickness measurement protocol*. An additional image was acquired for each plantar foot site; a single unmagnified, broad field-of-view image.

Ultrasound tissue characteristics were assessed for each anatomical layer of the plantar skin and fat pad (described in the *thickness measurement protocol*). Tissue characteristics assessed were *echogenicity* (the sonographic features of the tissue due to reflection of the ultrasound wave) and *definition* (how distinct the tissue or anatomical layer is). Assessments were scored using a dichotomous scoring scale; ‘*same’* or ‘*not-same*’; determined by comparing images against a reference set of foot images. The reference set included images representing each discrete anatomical layer at each foot site acquired from a healthy young adult foot. For intra-observer testing, one experienced sonographer (TM) independently analysed and scored the images. Scores were recorded at two-time points, one-week apart, on a custom designed worksheet using the same assessment environment (ambient lighting, monitor). For inter-observer testing, two experienced sonographers independently performed tissue characterisation assessments from the images without control of the assessment environment. Guidelines and visual examples on how to make the assessments were provided to observers in the form of a non-audio Microsoft PowerPoint slide show. Further detail on the assessment method is provided in S1 Fig.

### Statistical methods

Analyses were undertaken using Statistical Package for the Social Sciences (SPSS, IBM Corp. Released 2017. IBM SPSS Statistics for Macintosh, Version 25.0. Armonk, NY: IBM Corp.) and Microsoft Excel (v1810). Statistical significance was set at *p <* 0.05.

#### Thickness measurements

Paired samples t-tests were used to compare the difference between means for trial 1 and trial 2. Intra-class correlation coefficients (ICC, two-way random effects, single measure, absolute agreement) with 95% confidence intervals (95%CI) were calculated to determine relative reliability. A measurement was accepted as reliable if the ICC was greater than 0.75. Absolute reliability was determined using the standard error of measurement (SEM) and the SEM as a percentage of the mean (*SEM% = (SEM / mean) × 100*), where the mean is the mean for all test observations from trial 1 and trial 2. SEM and SEM% were used to determine the minimum change needed between repeat observations in an individual to detect an actual (real) change in tissue characteristics. SEM% values below 10% were considered sensitive for detecting small change [33] and were used to add context to the ICC values to avoid over emphasising the clinical usefulness of any acceptable relative reliability results.

#### Tissue characteristic assessments

Cohen’s kappa (k) measure of agreement was used to determine intra- and inter observer agreement for paired tissue characteristic assessments. Confidence intervals (95%CI), significance values and standard error of kappa (SE_*(k)*_) were also calculated [30]. The minimum acceptable level of agreement was determined *a priori* to be a kappa value of 0.61, thus any ultrasound characteristic assessments with a kappa value below this were deemed to be unreliable.

## Results

Summary characteristics of participants contributing to intra-and inter-observer testing are described in Table 1.

**Table 1:** Participant characteristics.

		Ultrasound Thickness Measurements		Tissue characteristic assessments			
				Intra-observer		Inter-observer	
		Non-diabetic (N = 9)	Diabetic (N = 6)	Non-diabetic (N = 9)	Diabetic (N = 13)	Non-diabetic (N = 13)	Diabetic (N = 9)
**Age (SD), years**		41.38 (± 11.5)	63.17 (± 11.7)	58.5 (± 26.1)	64.0 (± 18)	65.50 (± 20.1)	66.89 (± 15.4)
**Gender**	Male	6 (66.7%)	3 (50%)	4 (44%)	1 (8%)	5 (38.5%)	1 (11%)
	Female	3 (33.3%)	3 (50%)	5 (56%)	12 (92%)	8 (61.5%)	8 (89%)

N; sample size, SD; standard deviation.

### Thickness measurements (intra-observer reliability)

Results for comparisons between Trial 1 and Trial 2 are displayed in Tables 2–5.

**Table 2:** Results for intra-observer reliability testing of skin and fat pad thickness at the heel foot region.

Anatomical Region and measurement state	Anatomical Layer	Measurement	Mean (mm) ± SD	Difference in Means (mm)	P value	ICC (95% CI)	P value	SEM (95% CI) (mm)	SEM %
**Heel: Unloaded**	L1	M1	2.88 ± 0.43	0.02	0.803	0.809† (0.517, 0.932)	**<0.001** **	0.182 (-0.174, 0.538)	6.33
		M2	2.86 ± 0.40						
	L2	M1	1.28 ± 0.40	-0.08	0.460	0.410 (-0.113, 0.754)	0.061	0.267 (-0.257, 0.792)	20.27
		M2	1.36 ± 0.30						
	L3	M1	1.12 ± 0.28	-0.05	0.615	0.066 (-0.482, 0.555)	0.409	0.239 (-0.229, 0.706)	20.93
		M2	1.16 ± 0.21						
	L4	M1	11.59 ± 2.07	-0.08	0.561	0.968† (0.910, 0.989)	**<0.001** **	0.372 (-0.357, 1.100)	3.19
		M2	11.68 ± 2.08						
	TSD	M1	18.75 ± 2.56	-0.45	**0.015** *	0.960† (0.821, 0.988)	**<0.001** **	0.524 (-0.503, 1.550)	2.76
		M2	19.19 ± 2.68						
**Heel: Compressed**	L1	M1	2.33 ± 0.32	-0.02	0.729	0.836† (0.578, 0.942)	**<0.001** **	0.145 (-0.140, 0.431)	6.21
		M2	2.35 ± 0.40						
	L2	M1	0.86 ± 0.30	-0.06	0.284	0.690 (0.310, 0.883)	**<0.001** **	0.153 (-0.147, 0.452)	17.20
		M2	0.92 ± 0.25						
	L3	M1	0.83 ± 0.19	-0.14	**0.034** *	0.174 (-0.220, 0.579)	0.212	0.162 (-0.155, 0.478)	17.97
		M2	0.97 ± 0.16						
	L4	M1	4.91 ± 1.94	0.01	0.963	0.941† (0.832, 0.980)	**<0.001** **	0.481 (-0.461, 1.422)	9.79
		M2	4.90 ± 2.02						
	TSD	M1	11.00 ± 2.52	-0.32	0.075	0.963† (0.885, 0.988)	**<0.001** **	0.497 (-0.477, 1.471)	4.45
		M2	11.32 ± 2.65						

SD = standard deviation, CI = confidence interval, ICC = intra-class correlation coefficient (single measure, two-way random effects, absolute agreement), SEM = standard error of measurement, SEM% = within-subject standard deviation as a percentage of the mean, L1 = Layer one (skin), L2 = Layer two (superficial subcutaneous layer), L3 = Layer three (horizontal fibrous band / layer), L4 = Layer four (deep subcutaneous layer), TSD = Total soft-tissue depth, M1 = measurement one (trial 1), M2 = measurement 2 (trial 2), mm = millimetres.

* The mean difference is significant at the 0.05 level.

** The mean difference is significant at the <0.001 level.

† Acceptable agreement.

**Table 3:** Results for intra-observer reliability testing of skin and fat pad thickness at the lateral sesamoid foot region.

Anatomical Region and measurement state	Anatomical Layer	Measurement	Mean (mm) ± SD	Difference in Means (mm)	P value	ICC (95% CI)	P value	SEM (95% CI) (mm)	SEM %
**Lateral Sesamoid: Unloaded**	L1	M1	2.37 ± 0.36	0.05	0.576	0.600 (0.141, 0.846)	**0.008** *	0.242 (-0.232, 0.716)	10.31
		M2	2.32 ± 0.40						
	L2	M1	0.87 ± 0.32	-0.06	0.311	0.811† (0.536, 0.932)	**<0.001** **	0.151 (-0.145, 0.447)	16.81
		M2	0.93 ± 0.37						
	L3	M1	0.92 ± 0.24	-0.01	0.807	0.727† (0.353, 0.900)	**0.001** *	0.122 (-0.117, 0.360)	13.21
		M2	0.93 ± 0.23						
	L4	M1	5.84 ± 1.25	-0.53	0.002*	0.875† (0.337, 0.966)	**<0.001** **	0.507 (-0.487, 1.501)	8.30
		M2	6.37 ± 1.62						
	TSD	M1	11.85 ± 1.91	-0.37	0.074	0.925† (0.776, 0.975)	**<0.001** **	0.563 (-0.541, 1.667)	4.68
		M2	12.21 ± 2.20						
**Lateral Sesamoid: Compressed**	L1	M1	1.63 ± 0.30	-0.06	0.357	0.659 (0.252, 0.870)	**0.003** *	0.170 (-0.163, 0.504)	10.25
		M2	1.69 ± 0.28						
	L2	M1	0.32 ± 0.33	0.12	0.056	0.671 (0.258, 0.876)	**0.001** *	0.160 (-0.153, 0.472)	61.47
		M2	0.20 ± 0.22						
	L3	M1	0.66 ± 0.19	-0.04	0.471	0.238 (-0.307, 0.660)	0.193	0.146 (-0.140, 0.433)	21.63
		M2	0.70 ± 0.15						
	L4	M1	1.53 ± 0.68	-0.18	0.110	0.857† (0.622, 0.950)	**<0.001** **	0.300 (-0.288, 0.887)	18.50
		M2	1.71 ± 0.90						
	TSD	M1	5.60 ± 1.20	0.04	0.783	0.905† (0.740, 0.967)	**<0.001** **	0.397 (-0.381, 1.176)	7.12
		M2	5.56 ± 1.37						

SD = standard deviation, CI = confidence interval, ICC = intra-class correlation coefficient (single measure, two-way random effects, absolute agreement), SEM = standard error of measurement, SEM% = within-subject standard deviation as a percentage of the mean, L1 = Layer one (skin), L2 = Layer two (superficial subcutaneous layer), L3 = Layer three (horizontal fibrous band / layer), L4 = Layer four (deep subcutaneous layer), TSD = Total soft-tissue depth, M1 = measurement one (trial 1), M2 = measurement 2 (trial 2), mm = millimetres.

* The mean difference is significant at the 0.05 level.

** The mean difference is significant at the <0.001 level.

† Acceptable agreement.

**Table 4:** Results for intra-observer reliability testing of skin and fat pad thickness at the 2^nd^ sub-metatarsal head foot region.

Anatomical Region and measurement state	Anatomical Layer	Measurement	Mean (mm) ± SD	Difference in Means (mm)	P value	ICC (95% CI)	P value	SEM (95% CI) (mm)	SEM %
**2^nd^ Sub-Metatarsal Head: Unloaded**	L1	M1	2.41 ± 0.28	-0.05	0.687	0.181 (-0.382, 0.630)	0.261	0.297 (-0.285, 0.880)	12.21
		M2	2.46 ± 0.37						
	L2	M1	0.91 ± 0.40	0.03	0.751	0.501 (-0.014, 0.081)	**0.028** *	0.240 (-0.230, 0.710)	26.92
		M2	0.88 ± 0.27						
	L3	M1	0.89 ± 0.15	-0.04	0.386	0.351 (-0.174, 0.722)	0.093	0.109 (-0.105, 0.323)	12.04
		M2	0.92 ± 0.12						
	L4	M1	6.29 ± 1.63	-0.23	0.173	0.920† (0.783, 0.972)	**<0.001** **	0.451 (-0.433, 1.335)	7.05
		M2	6.52 ± 1.56						
	TSD	M1	13.95 ± 2.11	-0.23	**0.046** *	0.976† (0.915, 0.992)	**<0.001** **	0.323 (-0.310, 0.955)	2.30
		M2	14.18 ± 2.06						
**2^nd^ Sub-Metatarsal Head: Compressed**	L1	M1	1.75 ± 0.35	-0.10	0.132	0.675 (0.287, 0.876)	**0.001** *	0.175 (-0.168, 0.517)	9.72
		M2	1.85 ± 0.27						
	L2	M1	0.22 ± 0.25	0.02	0.620	0.826† (0.559, 0.938)	**<0.001** **	0.098 (-0.094, 0.291)	47.29
		M2	0.20 ± 0.22						
	L3	M1	0.75 ± 0.13	0.02	0.536	0.718 (0.344, 0.895)	**0.001** *	0.068 (-0.065, 0.201)	9.21
		M2	0.73 ± 0.12						
	L4	M1	1.87 ± 0.80	-0.07	0.574	0.841† (0.593, 0.943)	**<0.001** *	0.333 (-0.319, 0.984)	17.46
		M2	1.94 ± 0.87						
	TSD	M1	7.92 ± 1.20	-0.10	0.444	0.933† (0.817, 0.977)	**<0.001** *	0.339 (-0.325, 1.002)	4.25
		M2	8.02 ± 1.42						

SD = standard deviation, CI = confidence interval, ICC = intra-class correlation coefficient (single measure, two-way random effects, absolute agreement), SEM = standard error of measurement, SEM% = within-subject standard deviation as a percentage of the mean, L1 = Layer one (skin), L2 = Layer two (superficial subcutaneous layer), L3 = Layer three (horizontal fibrous band / layer), L4 = Layer four (deep subcutaneous layer), TSD = Total soft-tissue depth, M1 = measurement one (trial 1), M2 = measurement 2 (trial 2), mm = millimetres.

* The mean difference is significant at the 0.05 level.

** The mean difference is significant at the <0.001 level.

† Acceptable agreement.

**Table 5:** Results for intra-observer reliability testing of skin and fat pad thickness at the 3^rd^ sub-metatarsal head foot region.

Anatomical Region and measurement state	Anatomical Layer	Measurement	Mean (mm) ± SD	Difference in Means (mm)	P value	ICC (95% CI)	P value	SEM (95% CI) (mm)	SEM %
**3^rd^ Sub-Metatarsal Head: Unloaded**	L1	M1	2.57 ± 0.38	0.10	0.268	0.648 (0.240, 0.864)	**0.003** *	0.232 (-0.223, 0.688)	9.22
		M2	2.47 ± 0.41						
	L2	M1	1.01 ± 0.34	0.14	0.014*	0.752† (0.296, 0.916)	**<0.001** **	0.157 (-0.151, 0.465)	16.76
		M2	0.87 ± 0.29						
	L3	M1	0.93 ± 0.19	0.05	0.316	0.563 (0.107, 0.827)	**0.011** *	0.119 (-0.114, 0.353)	13.16
		M2	0.88 ± 0.17						
	L4	M1	5.20 ± 1.89	-0.27	0.054	0.953† (0.846, 0.985)	**<0.001** **	0.390 (-0.374, 1.154)	7.31
		M2	5.47 ± 1.71						
	TSD	M1	12.66 ± 2.00	0.02	0.908	0.953† (0.867, 0.984)	**<0.001** **	0.420 (-0.403, 1.244)	3.32
		M2	12.64 ± 1.88						
**3^rd^ Sub-Metatarsal Head: Compressed**	L1	M1	1.80 ± 0.28	-0.08	0.240	0.638 (0.226, 0.860)	**0.003** *	0.187 (-0.180, 0.554)	10.18
		M2	1.88 ± 0.34						
	L2	M1	0.11 ± 0.15	0.02	0.528	0.757† (0.420, 0.911)	**<0.001** **	0.078 (-0.075, 0.230)	75.27
		M2	0.09 ± 0.17						
	L3	M1	0.70 ± 0.17	-0.05	0.365	0.304 (-0.223,0.695)	0.128	0.134 (-0.129, 0.397)	18.44
		M2	0.75 ± 0.15						
	L4	M1	1.57 ± 0.77	-0.16	0.220	0.812† (0.539, 0.932)	**<0.001** **	0.356 (-0.342,1.054)	21.48
		M2	1.74 ± 0.88						
	TSD	M1	7.17 ± 1.07	0.08	0.761	0.690 (0.285, 0.885)	**0.002** *	0.721 (-0.692, 2.135)	10.12
		M2	7.08 ± 1.52						

SD = standard deviation, CI = confidence interval, ICC = intra-class correlation coefficient (single measure, two-way random effects, absolute agreement), SEM = standard error of measurement, SEM% = within-subject standard deviation as a percentage of the mean, L1 = Layer one (skin), L2 = Layer two (superficial subcutaneous layer), L3 = Layer three (horizontal fibrous band / layer), L4 = Layer four (deep subcutaneous layer), TSD = Total soft-tissue depth, M1 = measurement one (trial 1), M2 = measurement 2 (trial 2), mm = millimetres.

* The mean difference is significant at the 0.05 level.

** The mean difference is significant at the <0.001 level.

† Acceptable agreement.

Relative reliability was achieved for unloaded measurements at the plantar heel for L1 (ICC 0.81), L2 (ICC 0.97) and TSD (ICC 0.96). These measurements demonstrated SEMs ranging from 0.18–0.52mm representing a 2.76%– 6.33% test-retest error as a percentage of the mean. Relative reliability was achieved for compressed measurements at the plantar heel for L1 (ICC 0.84), L4 (ICC 0.94) and TSD (ICC 0.96). These measurements demonstrated SEMs ranging from 0.15–0.50mm, representing a 4.45%– 9.79% test-retest error as a percentage of the mean.

Relative reliability was achieved for unloaded measurements at the lateral sesamoid for L2 (ICC 0.81), L4 (ICC 0.88) and for TSD (ICC 0.93). These measurements demonstrated SEMs ranging from 0.15–0.56mm, representing a 4.68%– 16.81% test-retest error as a percentage of the mean. Relative reliability was achieved for compressed measurements at the lateral sesamoid for L4 (ICC 0.86) and TSD (ICC 0.91). These measurements demonstrated SEMs ranging from 0.30–0.40mm, representing a 7.12%– 18.5% test-retest error as a percentage of the mean.

Relative reliability was achieved for unloaded measurements at the second metatarsal head for L4 (ICC 0.98) and TSD (ICC 0.92). These measurements demonstrated SEMs ranging from 0.32–0.45mm representing a 2.30%– 7.05% test-retest error as a percentage of the mean. Relative reliability was achieved for compressed measurements at the second metatarsal head for L2 (ICC 0.83), L4 (ICC 0.84) and for TSD (ICC 0.93). These measurements demonstrated SEMs ranging from 0.10–0.34mm, representing a 4.25%– 47.29% test-retest error as a percentage of the mean.

Relative reliability was achieved for unloaded measurements at the third metatarsal head for L2 (ICC 0.75), L4 (ICC 0.95) and TSD (ICC 0.95). These measurements demonstrated SEMs ranging from 0.16–0.42mm, representing a 3.32%– 6.76% test-retest error as a percentage of the mean. Relative reliability was achieved for compressed measurements at the third metatarsal head for L2 (ICC 0.76) and L4 (ICC 0.81). These measurements demonstrated SEMs ranging from 0.08–0.36mm, representing a 21.48–75.27% test-retest error as a percentage of the mean.

### Tissue characteristic assessments (intra- and inter-observer agreement)

Results of testing intra- and inter-observer agreement for tissue characteristic assessments are described in Table 6. Categorical data is presented in S1, S2 Appendices.

**Table 6:** Results of inter- and intra-observer agreement for skin and fat pad tissue characteristic assessments.

	Measurement	Kappa Value (k)	95% CI	SE(k)	Sig. (p-value)
**Intra-observer**	Heel L1	0.70†	0.381, 1.013	0.161	**0.001** **
	Heel L2-4	0.38	0.043, 0.725	0.174	0.045
	LS L1	0.52	0.157, 0.887	0.186	**0.014** *
	LS L2-4	0.70†	0.319, 1.079	0.194	**0.001** **
	MTH2 L1	0.80†	0.545, 1.063	0.132	**0.00002** **
	MTH2 L2-4	0.70†	0.405, 1.001	0.152	**0.001** **
	MTH3 L1	0.90†	0.707, 1.091	0.098	**0.00002** **
	MTH3 L2-4	0.79†	0.523, 1.061	0.137	**0.0001** **
**Inter-observer**	Heel L1	0.32	-0.356, 0.495	0.217	0.124
	Heel L2-4	0.05	-0.365, 0.383	0.191	0.806
	LS L1	0.37	-0.300, 0.441	0.189	0.690
	LS L2-4	0.25	-0.348, 0.452	0.204	0.225
	MTH2 L1	0.21	-0.324, 0.401	0.185	0.277
	MTH2 L2-4	0.24	-0.377, 0.481	0.219	0.262
	MTH3 L1	0.45	-0.288, 0.460	0.191	**0.035** *
	MTH3 L2-4	0.17	-0.381, 0.454	0.213	0.416

L1; Layer one (skin), L2-4; Layer two, three and four (superficial subcutaneous microchamber layer (L2), horizontal fibrous band (L3) and deep subcutaneous macrochamber layer (L4)), TSD; Total soft-tissue depth, LS; lateral sesamoid fat pad region, MTH2; 2nd sub-metatarsal head fat pad, MTH3; 3rd sub-metatarsal head fat pad. Sig.; statistical significance

* ***p <* 0.05,**

** ***p <* 0.001.**

† criteria for accepting agreement.

Intra-observer agreement was accepted for tissue characteristic assessments made at the heel for L1 (k 0.70), at the lateral sesamoid for layers 2–4 (k 0.70), at the second metatarsal head for L1 (k 0.80) and layers 2–4 (k 0.70), and at the third metatarsal head for L1 (k 0.90) and layers 2–4 (k 0.79).

Acceptable inter-observer agreement was not demonstrated for any tissue characteristic assessments.

## Discussion

This study tested the reliability and agreement of ultrasound thickness measurements and tissue characterisation assessments of the plantar skin and the discrete plantar fat pad layers in a cohort of people with and without diabetes. Measurements and assessments were made at plantar foot sites where the tissues are at risk of ulceration in diabetic populations. Prior to this study, ultrasound thickness measurements of the discrete fat pad anatomical layers and tissue characterisation assessments of the plantar fat pad and skin tissue have not been assessed for reliability.

Thickness measurements made in the unloaded state achieved intra-observer reliability at all sites for both the macrochamber layer (layer 4) and total soft-tissue depth (TSD), providing a reliable suite of ultrasound measurements to assess the plantar soft tissues of the foot that are at risk of soft-tissue injury in people with diabetes. This was achieved despite some challenges when making measurements due to anatomical features such as cortical irregularity of the calcaneus, plantar fasciopathy and enthesophytes, and application of appropriate transducer pressure that was needed to acquire an artefact free image. Relative reliability for TSD measurements at the heel pad, made in the unloaded state (ICC 0.96) were similar to reliability results reported by Telfer et. al. [34] (ICC 0.94–0.95) who measured heel fat pad thickness (excluding the skin) at a similar site used in our study, but measured during gait using an in-shoe transducer. The consistently lower absolute reliability values for unloaded TSD measurements (SEM%; 2.3%–4.68%), compared to unloaded measurements of the microchamber layer (SEM%; 3.19%–8.3%), indicate that unloaded measurements of TSD are more sensitive for detecting small changes.

Absolute reliability for measurements of the macrochamber layer thickness and TSD was always poorer when the measurement was made in the compressed state (SEM%; 4.25%–21.48%), compared to the unloaded state (SEM%; 2.3%–8.3%). Compressed TSD measurements at the metatarsal heads demonstrated similar or reduced absolute reliability (SEM%; 4.25%–10.12%) compared to a prior investigation [35] reporting a coefficient of variation of 10% for weight bearing TSD measurements at the metatarsal heads. Reduced reliability for measurements made in compressed states may be attributable to the compression method used, in which hand-held (manual) compression was applied to the tissues through the transducer. During the compression, the transducer may drift from the central measurement position creating measurement variations between trials. This drift may be more pronounced for measurements made at the forefoot, where the small, round shape of the metatarsal heads and sesamoids, compared to the broader and flatter calcaneum, increases the risk of the transducer slipping from a central measurement position.

The findings of acceptable reliability for measurements of fat pad thickness can be used to build on the findings of prior studies. Kumar et. al. [23] explored the sub-metatarsal head fat pad thickness in people with and without diabetes using a similar ultrasound approach to that used for measuring unloaded thickness of layer 4 in this study. They reported a reduction in the fat pad thickness for people with diabetes and recommended using ultrasound to screen the feet of people with diabetes to prevent foot complications. The present study has shown that unloaded measurements of layer 4, like those used in the Kumar et. al. study, are reliable and can be used to detect differences in fat pad thickness between groups. Importantly, however, the present study has demonstrated that measurements of the *total tissue depth* are the most sensitive for detecting small change, and thus, may be the preferred measurement method for detecting change in individuals across repeated measurements.

Plantar skin thickness measurements demonstrated intra-observer reliability only when made at the site where the skin was the thickest; at the heel pad (ICC 0.81–0.84). It is possible that at the other sites where the skin was thinner, measurement became more difficult and more susceptible to error. This has implications where significant differences have been reported for measurements of skin thickness at the metatarsal heads between people with and without diabetes [23]. Our finding of poor intra-observer reliability for these measurements indicate that these reports should be interpreted with caution. Duffin et.al. [36] was able to achieve a higher ICC value (0.92) compared to our results, for skin thickness measured at the first metatarsophalangeal joint. However, their measurements were made in a paediatric population (with and without diabetes), and their method methodology was not described in detail enough to make comparisons.

The superficial subcutaneous layer (layer 2) and the horizontal fibrous band (layer 3) were not consistently reliable across all sites. This may be explained by the small magnitude of these structures, and similar to skin measurements, are difficult to measure and susceptible to measurement error. Non-uniform structure and poor differentiation may also have contributed. Layer 3 is a non-linear and non-uniform structure with surface undulations, and on ultrasound demonstrated a discontinuous mesh-like configuration creating difficulties in making judgements on where to measure. The surface inconsistencies of layer 3 potentially created similar measurement difficulties for layer 2, which opposes layer 3 on its deep surface. Layer 2 is also difficult to differentiate from other structures when viewed on ultrasound images, particularly in the forefoot. The reliability and validity to use the measurements of these structures clinically may be improved with the use of a higher frequency broadband transducer and high-definition equipment.

Assessments of ultrasound tissue characteristics can be useful adjuncts to size measurements, by providing more information about anatomical states and the extent of any tissue injury, structural change or pathology that is present. When structural change manifests only as tissue alterations, relying on thickness measurements alone could falsely lead to the assumption of structural normality. There appear to be no prior studies that specifically evaluate the intra- and inter-observer agreement of ultrasound tissue assessments of the plantar skin and individual anatomical layers of the fat pad. Intra-observer agreement was demonstrated for all tissue characterisation assessments, which included echogenicity and definition, excepting for the fat pad (layers 2–4) at the heel, and the skin (layer 1) at the lateral sesamoid foot region. We did not demonstrate inter-observer agreement for any tissue characterisation assessments, supporting the view that assessments of ultrasound tissue characteristics of musculoskeletal structures are highly subjective and variable [26–28], even when assessed by experienced sonographers using defined protocols. Therefore, these assessments have limited clinical applicability where multiple sonographers may be involved. Our findings support the recommendation that serial ultrasound tissue assessments of musculoskeletal structures on the same patient over time are better made by the same observer where possible [37, 38].

If inter-observer agreement could be improved, it would make assessments of ultrasound tissue characteristics more valid for clinical application. This could be achieved with alternate assessment methods to the ones used in this study. In this study static images were used to make the tissue characterisation assessments. Inter-observer agreement may be improved by making the assessments from real-time images at the time of assessment, rather than retrospectively from static images. Naredo et.al. [28] report moderate to good results for inter-observer agreement using real-time ultrasound imaging to evaluate ultrasound characteristics of various musculoskeletal conditions. Real-time imaging (by using multiple dynamic scanning manoeuvres to image in multiple planes) aids a three- or four-dimensional understanding of the structure and its inherent acoustic properties. The sonographer will also have a better knowledge of technical parameters used and the technical challenges encountered due to variations in acoustic properties in the feet of individuals and can take these into account when making decisions about tissue characteristics in real-time. The attenuating properties of an individual’s foot are an example. Cheng et. al. [39], in a study evaluating the plantar fascia using ultrasound imaging, reported that agreement was affected by attenuation of the ultrasound beam from the plantar skin, particularly at the heel. Similarly, this current study, demonstrated only fair intra-observer and slight inter-observer agreement for ultrasound tissue characteristic assessments of the heel fat pad. Limiting the effect of attenuation is problematic as it is an inherent tissue property. Adjustment of image settings (such as adjusting gain or frequency) during image acquisition can regulate image degradation due to attenuation, but these adjustments may also introduce variations in image parameters across individuals, potentially contributing to assessment variations between observers.

The dichotomous scoring method to make subjective assessments of tissue characteristics may have also influenced agreement. The choice of the scoring method was predicated on previous ultrasound studies where subjective assessments demonstrated good inter-observer agreement. These studies used dichotomous scales to make decisions on the ‘presence’ or ‘absence’ of pathology such as joint effusion [28, 40, 41], tendon disease or injury [28, 42, 43] distended bursa [28]. However, in the setting of the current study, multiple and sometimes subtle ultrasound features needed to be considered to make a decision. The dichotomous scale may have been too broad to capture and describe these features, making decision making difficult and influencing the low inter-observer agreement. Other studies investigating differences in ultrasound tissue characteristics of musculoskeletal structures have also reported low agreement [28, 37, 39].

Inter-observer agreement may have been improved by controlling the environment in which the assessments were made. Environmental factors that could be controlled include monitor distance from the observer, the display calibration, the monitor design and the size of the images, monitor brightness and contrast settings and ambient room lighting [44]. In this study, each observer assessed images in an uncontrolled environment, using their personal computing resources and environments. This was an intentional part of the study design to gain an understanding of how well the imaging modality performed in an unrestrictive environment.

Inter-observer agreement is reported to improve with training in the interpretation and application of an imaging protocol [45, 46]. Thus, structured training, precise protocol terminology and simplified instructions may also help to reduce interpretation subjectiveness and improve consistency.

Sub-optimal imaging in some participants may have contributed to low inter-observer agreement. Sub-optimal images can result in assessment errors due to lack of image clarity. In this study, the sonographer was not restricted by predetermined technical settings and was permitted to make technical adjustments for each participant to optimise the images. However, the sonographer was restricted to a single 12MHz broadband transducer which standardised the sonographic tissue detail, image quality and field of view [47, 48]. This standardisation may have resulted in reduced image quality in some participants. An alternate approach may be to allow the operator to choose the transducer and frequency they believe is most appropriate for each individual.

The use of grey scale analysis software for analysing ultrasound tissue characterisation of the plantar tissues may improve inter-observer agreement. This process uses a histogram to quantify tissue echogenicity [49] and may reduce subjectivity of echogenicity interpretations. Image analysis software would require that the imaging process be highly standardised with fixed imaging equipment and scanning parameters (gain, dynamic range, frequency, focal zone, depth settings transducer tilt), to avoid influencing the histogram analysis and producing varying or inaccurate echogenicity results [50]. This standardisation limits clinical applicability, where it is best practice to adjust ultrasound imaging technical parameters in real-time to respond to tissue variations in individuals and optimise images.

Measurements of plantar soft tissue thickness and assessments of plantar tissue characteristics demonstrating intra-observer reliability and agreement in this study may be useful to the diabetic population and clinicians who treat them. A number of studies have demonstrated that ultrasound measurements of the thickness of plantar soft tissues are different between people with and without diabetes, suggesting that such measurements may have a role in identifying causal or predictive changes in the feet of diabetics [25]. The measurements may also be useful in non-diabetic populations, such as those who have undergone foot reconstruction where localised high plantar pressure is suspected.

Further investigations can be made using a suite of measurements and assessments that have demonstrated intra-observer reliability and agreement in this study.

At sites which have not shown ultrasound reliability or agreement, an alternative imaging method could include magnetic resonance imaging (MRI) which has been shown to demonstrate structural changes in the plantar foot of people with diabetes [51]. Reliability of MRI measurements of the plantar fat pad at various sites has been demonstrated [52], but the reliability of MRI measurements of plantar skin thickness, and assessments of plantar tissue characteristics in the foot with diabetes has not yet been reported and would require investigation.

## Conclusion

An ultrasound imaging protocol using thickness measurements and assessments of tissue characteristics of the plantar skin and plantar fat pad was tested for its reliability.

Measurements of the total soft-tissue thickness made in the unloaded state demonstrate intra-observer reliability and are the most sensitive for detecting small changes on repeated measures.

Assessments of tissue characteristics for the skin at the heel, the fat pad at the lateral sesamoid region and both the skin and the fat pad at the heads of the second and third metatarsals demonstrated intra-observer reliability. No ultrasound tissue characteristic assessment demonstrated inter-observer reliability.

This study does not address inter-observer reliability of ultrasound measurements of the thickness of plantar foot structures and did not demonstrate inter-observer agreement for ultrasound assessments of tissue characteristics, and therefore, caution should be applied when using these measurements, using them in situations where repeated assessments are performed by the same operator or observer.

## Supporting information

S1 Fig Details of ultrasound tissue characteristic assessments for score categorisation. (DOCX)

S1 Appendix Intra-observer categorical data. (DOCX)

S2 Appendix Inter-observer categorical data. (DOCX)
