# Supplementary material for: Reliability of ultrasound in evaluating the plantar skin and fat pad of the foot in the setting of diabetes
Source: PLoS One. 2021 Sep 23;16(9):e0257790. doi: 10.1371/journal.pone.0257790 (PMC8459958; doi:10.1371/journal.pone.0257790)
Supplement: S1 Fig — (DOCX) [file pone.0257790.s001.docx]

**S1 Fig.** Details of ultrasound tissue characteristic assessments for score categorisation.

| **Anatomical Layer** | **Description of tissue characteristics (*echogenicity* and *definition*)** | |
| --- | --- | --- |
|  | Score = ‘***same***’ | Score = ‘***not-same****’* |
| **L1**  (the plantar skin: combined epidermis and dermis) | Demonstrates characteristics present on the reference images: echogenic and bi-laminar epidermis, homogenous and hyperechoic (bright) dermis when compared to the more hypoechoic (darker) hypodermis. | Conditions in middle column (‘*same’*) are not met. |
| **L2-L4**  (superficial subcutaneous microchamber layer, horizontal fibrous layer, deep subcutaneous macrochamber layer) | Demonstrates characteristics present on the reference images: a definable hypoechoic and heterogeneous microchamber layer (L2), an echogenic and defined horizontal fibrous layer (L3) and a deeper macrochamber layer (L4) that appeared heterogeneous with a hypoechoic background. At least five adipocyte macrochambers should be definable with distinguishable fibroelastic septa, of a similar thickness and definition, to that displayed in the reference images. |  |

L1; plantar skin, L2-4; plantar fat pad (L2 microchamber layer, L3 horizontal fibrous layer, L4 macrochamber layer).
