# Supplementary material for: Reliability of ultrasound in evaluating the plantar skin and fat pad of the foot in the setting of diabetes
Source: PLoS One. 2021 Sep 23;16(9):e0257790. doi: 10.1371/journal.pone.0257790 (PMC8459958; doi:10.1371/journal.pone.0257790)
Supplement: S1 Appendix — (DOCX) [file pone.0257790.s002.docx]

S1 Appendix Intra-observer scores for categorical data

| ID | DB STATUS | Heel L1 Obs1 | Heel L1 Obs2 | Heel L2-4 Obs1 | Heel L2-4 Obs2 | LS L1 Obs1 | LS L1 Obs2 | LS L2-4 Obs1 | LS L2-4 Obs2 | MTH2 L1 Obs1 | MTH2 L1 Obs2 | MTH2 L2-4 Obs1 | MTH2 L2-4 Obs2 | MTH3 L1 Obs1 | MTH3 L1 Obs2 | MTH3 L2-4 Obs1 | MTH3 L2-4 Obs2 |
| --- | --- | --- | --- | --- | --- | --- | --- | --- | --- | --- | --- | --- | --- | --- | --- | --- | --- |
| 2 | DBM | 2 | 2 | 2 | 2 | 2 | 2 | 2 | 2 | 1 | 1 | 2 | 2 | 1 | 1 | 2 | 2 |
| 3 | DBM | 2 | 2 | 2 | 2 | 2 | 2 | 2 | 2 | 2 | 2 | 2 | 2 | 2 | 2 | 2 | 2 |
| 9 | NDB | 1 | 1 | 2 | 2 | 1 | 2 | 2 | 2 | 1 | 1 | 2 | 2 | 1 | 1 | 2 | 2 |
| 14 | DBM | 2 | 2 | 2 | 2 | 2 | 1 | 2 | 2 | 2 | 2 | 2 | 2 | 2 | 2 | 2 | 2 |
| 17 | DBM | 1 | 1 | 2 | 2 | 1 | 1 | 2 | 2 | 1 | 1 | 2 | 2 | 2 | 2 | 2 | 2 |
| 25 | DBM | 2 | 2 | 2 | 2 | 1 | 1 | 2 | 2 | 2 | 2 | 2 | 2 | 1 | 1 | 2 | 2 |
| 26 | NDB | 1 | 2 | 1 | 2 | 2 | 2 | 2 | 2 | 2 | 2 | 2 | 2 | 2 | 2 | 2 | 2 |
| 30 | DBM | 2 | 2 | 2 | 2 | 2 | 2 | 2 | 2 | 2 | 1 | 2 | 2 | 2 | 2 | 2 | 2 |
| 33 | DBM | 1 | 1 | 2 | 2 | 1 | 1 | 1 | 2 | 1 | 2 | 1 | 2 | 2 | 2 | 2 | 2 |
| 36 | DBM | 1 | 1 | 1 | 2 | 1 | 1 | 2 | 2 | 1 | 1 | 2 | 2 | 1 | 1 | 2 | 2 |
| 39 | NDB | 1 | 1 | 2 | 1 | 1 | 2 | 2 | 2 | 1 | 1 | 1 | 1 | 1 | 1 | 2 | 2 |
| 42 | NDB | 1 | 2 | 1 | 1 | 2 | 2 | 2 | 2 | 2 | 2 | 1 | 2 | 1 | 1 | 1 | 2 |
| 46 | NDB | 1 | 1 | 1 | 1 | 1 | 1 | 1 | 2 | 1 | 1 | 2 | 2 | 1 | 1 | 2 | 2 |
| 47 | NDB | 1 | 1 | 1 | 2 | 2 | 2 | 2 | 2 | 1 | 1 | 2 | 2 | 1 | 1 | 1 | 1 |
| 48 | NDB | 1 | 1 | 1 | 2 | 2 | 2 | 1 | 1 | 1 | 1 | 1 | 1 | 1 | 1 | 1 | 1 |
| 50 | DBM | 1 | 1 | 1 | 2 | 2 | 1 | 2 | 2 | 1 | 1 | 1 | 1 | 1 | 1 | 1 | 1 |
| 55 | NDB | 1 | 1 | 1 | 1 | 1 | 1 | 1 | 1 | 1 | 1 | 1 | 1 | 1 | 1 | 1 | 1 |
| 58 | NDB | 2 | 2 | 2 | 2 | 2 | 2 | 2 | 2 | 2 | 2 | 1 | 1 | 2 | 2 | 1 | 1 |
| 60 | DBM | 1 | 1 | 1 | 2 | 1 | 2 | 2 | 2 | 1 | 1 | 1 | 2 | 1 | 2 | 2 | 2 |
| 62 | DBM | 2 | 1 | 1 | 1 | 2 | 2 | 1 | 1 | 1 | 1 | 1 | 1 | 1 | 1 | 1 | 2 |
| 63 | DBM | 1 | 1 | 1 | 1 | 2 | 2 | 2 | 2 | 1 | 1 | 2 | 2 | 1 | 1 | 1 | 1 |
| 67 | DBM | 1 | 1 | 1 | 1 | 2 | 2 | 2 | 2 | 2 | 2 | 2 | 2 | 1 | 1 | 2 | 2 |

ID; participant identification number, DB; diabetes, DBM; diabetes mellitus, NDB; non-diabetic, L1; plantar skin, L2-4; fat pad (superficial microchamber layer, horizontal fibrous band, deep macrochamber layer), LS; lateral sesamoid foot region, MTH2; second metatarsal head foot region, MTH3; third metatarsal head foot region, Obs1; observation one, Obs2; observation two. 1; scored *same* as reference foot, 2; scored *not same* as reference foot.
